# Supplementary figures and images for: IL-1-conferred gene expression pattern in ERα+ BCa and AR+ PCa cells is intrinsic to ERα− BCa and AR− PCa cells and promotes cell survival
Source: BMC Cancer. 2020 Jan 20;20:46. doi: 10.1186/s12885-020-6529-9 (PMC6971947; doi:10.1186/s12885-020-6529-9)

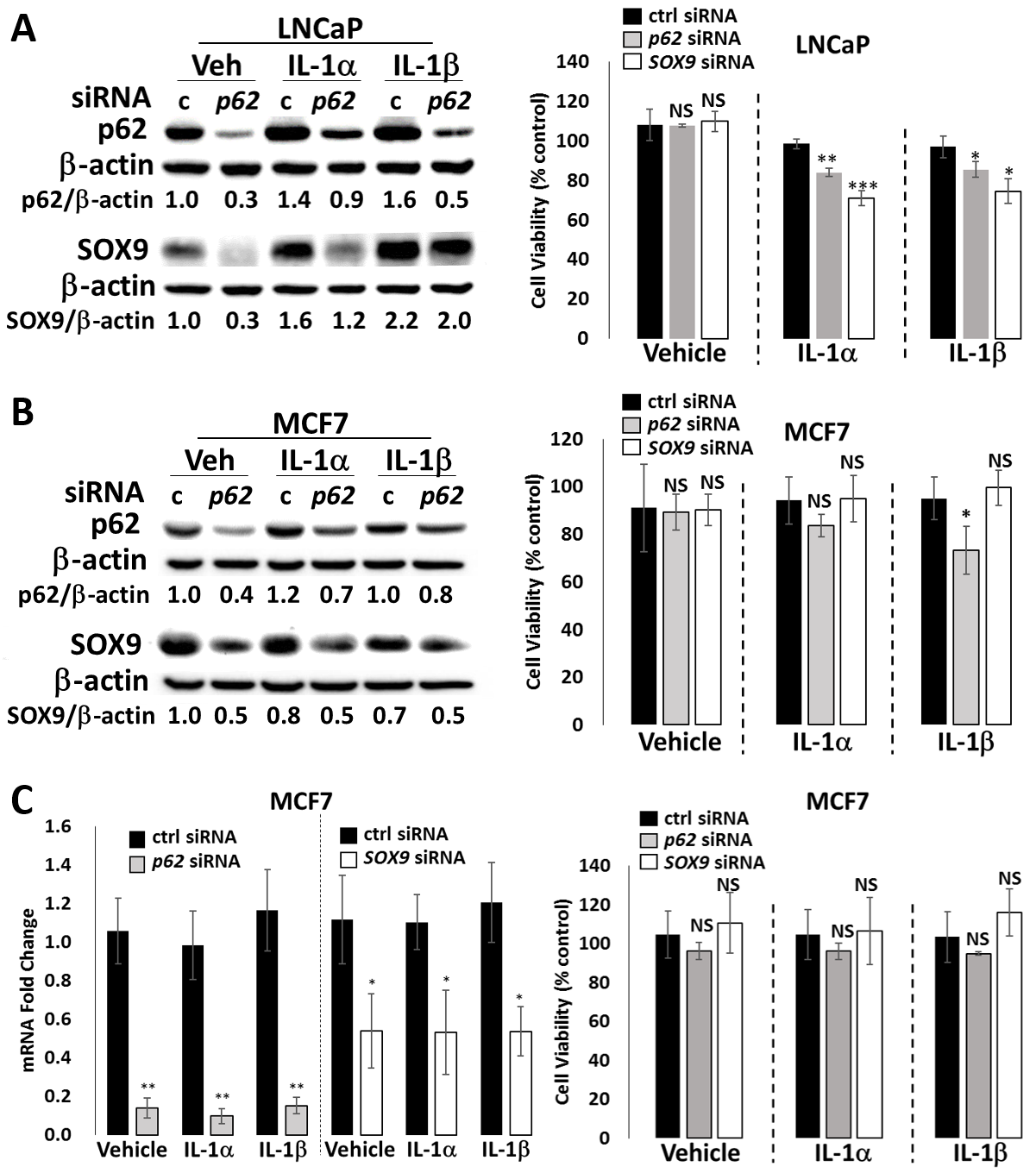

Supplement: Supplementary file 1 — Additional file 1: Figure S1. p62 and/or SOX9 are not required for cell survival in HR+ PCa and BCa cell lines. (A) LNCaP and (B) MCF7 cell lines were treated with 70 nM control siRNA (“c”), p62 siRNA, or SOX9 siRNA and after 1 day in siRNA, cells were treated with vehicle control (“veh”) or 25 ng/ml IL-1 for an additional 3 days. Western blot was performed to confirm p62 or SOX9 knockdown and MTT was performed to determine cell viability. (C) MCF7 cell lines were treated with 70 nM control siRNA, p62 siRNA, or SOX9 siRNA and after 1 day in siRNA, cells were treated with vehicle control or 25 ng/ml IL-1 for an additional 5 days. siRNA is transient; therefore, an additional 70 nM siRNA was added on day 4 after the initial siRNA treatment. RT-qPCR was performed to validate p62 or SOX9 knockdown and MTT was performed to determine cell viability. Loss of p62 or SOX9 is slightly cytotoxic for IL-1-treated LNCaP cell but is not cytotoxic for IL-1-treated MCF7 cells. N = 3 biological replicates; error bars, +/−STDEV; p-value, * ≤ 0.05, ** ≤ 0.005, *** ≤ 0.0005. mRNA fold change and cell viability are normalized to control siRNA for each treatment. Western blot densitometry is shown. [file 12885_2020_6529_MOESM1_ESM.tif]

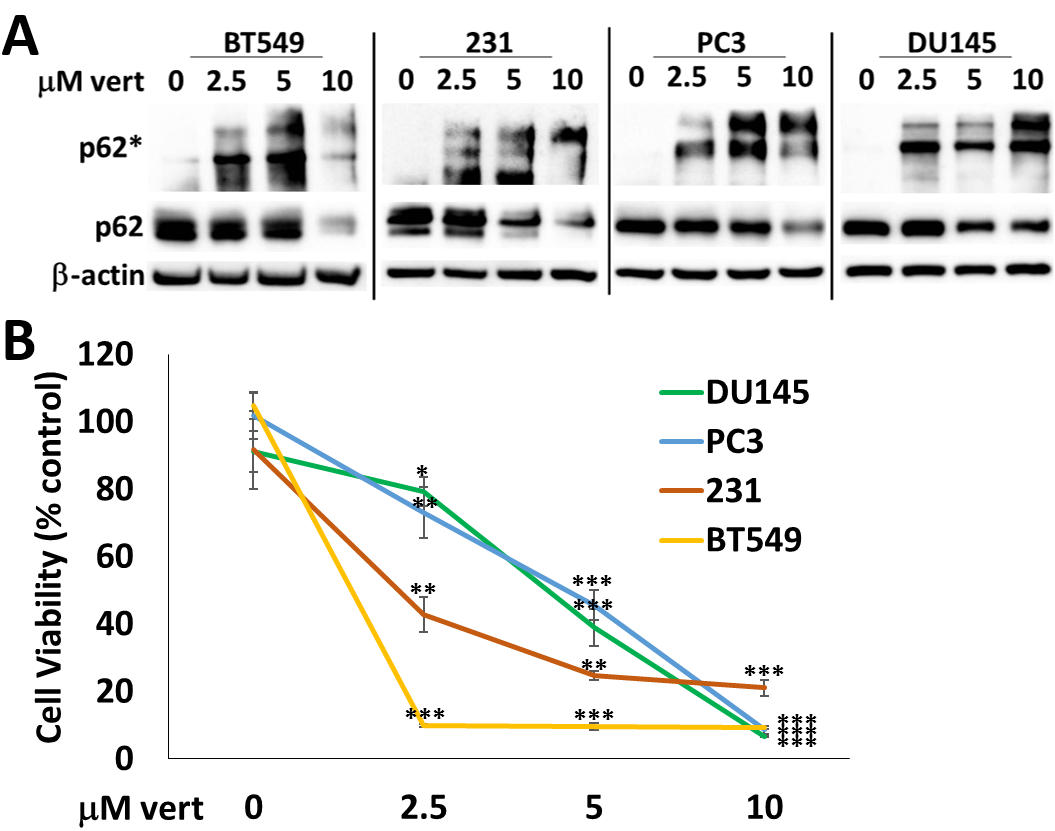

Supplement: Supplementary file 2 — Additional file 2: Figure S2. Verteporfin is cytotoxic for HR− BCa and PCa cell lines. (A) MDA-MB-231, BT549, PC3, and DU145 cell lines were treated with vehicle control, 2.5, 5, or 10 μM verteporfin for 1 day. Western blot analysis shows oligomerized p62 (p62*), indicating treatment efficacy. (B) Cells were treated with vehicle control, 2.5, or 5, 10 μM verteporfin for 3 days and MTT assay was performed to assess cell viability. Verteporfin is cytotoxic for MDA-MB-231, BT549, PC3, and DU145 cell lines. N = 3 biological replicates; error bars, +/−STDEV; p-value, ** ≤ 0.005, *** ≤ 0.0005. Cell viability is normalized to vehicle control for each cell line. [file 12885_2020_6529_MOESM2_ESM.tif]
